# STAT3 regulates cytotoxicity of human CD57+ CD4+ T cells in blood and lymphoid follicles

**DOI:** 10.1038/s41598-018-21389-8

**Published:** 2018-02-23

**Authors:** Jalila Alshekaili, Rochna Chand, Cindy Eunhee Lee, Susan Corley, Kristy Kwong, Ilenia Papa, David A. Fulcher, Katrina L. Randall, Jennifer W. Leiding, Cindy S. Ma, Marc R. Wilkins, Gulbu Uzel, Chris C. Goodnow, Carola G. Vinuesa, Stuart G. Tangye, Matthew C. Cook

**Affiliations:** 1 Department of Immunology and Infectious Disease, John Curtin School of Medical Research, Australian National University, ACT, 2601 Australia; 2 Translational Research Unit, Level 6 Building 10, Canberra Hospital, Woden, ACT 2606 Australia; 3 Department of Immunology, Canberra Hospital, Woden, ACT 2606 Australia; 4 Systems Biology Initiative, School of Biotechnology and Biomolecular Science, University of New South Wales, Sydney, Australia; 5 Division of Allergy, Immunology, and Rheumatology, Department of Pediatrics, University of South Florida and Johns Hopskins All Children’s Hospital, St Petersburg, Florida USA; 6 Immunology Division, Garvan Institute of Medical Research, 384 Victoria Street, Darlinghurst, NSW 2010 Australia; 7 Laboratory of Clinical Infectious Diseases, National Institute of Allergy and Infectious Diseases, National Institutes of Health, Bethesda, Maryland USA; 8 St Vincent’s Clinical School, Faculty of Medicine, University of New South Wales, Darlinghurst, NSW 2010 Australia; 9 Present Address: Department of Microbiology and Immunology, Sultan Qaboos University Hospital, Seeb, Oman

## Abstract

A subset of human follicular helper T cells (TFH) cells expresses CD57 for which no distinct function has been identified. We show that CD57+ TFH cells are universally PD-1^hi^, but compared to their CD57− PD-1^hi^ counterparts, express little IL-21 or IL-10 among others. Instead, CD57 expression on TFH cells marks cytotoxicity transcriptional signatures that translate into only a weak cytotoxic phenotype. Similarly, circulating PD-1+ CD57+ CD4+ T cells make less cytokine than their CD57− PD-1+ counterparts, but have a prominent cytotoxic phenotype. By analysis of responses to STAT3-dependent cytokines and cells from patients with gain- or loss-of-function *STAT3* mutations, we show that CD4+ T cell cytotoxicity is STAT3-dependent. TFH formation also requires STAT3, but paradoxically, once formed, PD-1^hi^ cells become unresponsive to STAT3. These findings suggest that changes in blood and germinal center cytotoxicity might be affected by changes in STAT3 signaling, or modulation of PD-1 by therapy.

## Introduction

Human follicular helper T (TFH) cells are characterized by high expression of CXCR5^1–6^, PD-1 and ICOS, and abundant production of IL-21, which is important for B cell help and antibody production^5,7–11^. TFH cells are heterogeneous for phenotype and function. In humans, but not mice, a significant subset of TFH cells expresses CD57^6^. There is conflicting evidence with regard to the relative propensity of the CD57+ and CD57− subsets to provide help to B cells^12,13^, and to date there is no other evidence that this CD57+ subset is functionally distinct. While the function of CD57+ TFH cells remains obscure, other evidence indicates that both CD57 and PD-1 are expressed by exhausted circulating T cells, characterised by proliferative incompetence and reduced cytokine production^14,15^. Indeed, high level PD-1 expression is observed on CD8+ T cells after chronic antigen stimulation and marks cells in a state of clonal exhaustion^16–18^. CD57 is also expressed on a subset of terminally differentiated NK cells with attenuated responsiveness to cytokines but have cytotoxic ability that is induced by IL-2^19,20^.

TFH cells are largely confined to secondary lymphoid organs, but there is evidence that CXCR5+ or PD-1+ CD4 T cells in the blood are a circulating counterpart of TFH cells (cTFH)^21–23^. Importantly, the proportion of cTFH cells correlates with disease activity in various autoimmune diseases, including SLE, juvenile dermatomyositis and rheumatoid arthritis^21,23–25^. Furthermore, in patients with HIV infection, abundance of PD-1+ CD4+ T cells in the blood correlates with titers of neutralizing antibodies^26^.

Both TFH and cTFH express PD-1 with or without CD57. We set out to determine if these subsets shared functions, and if these were distinct from their CD57− counterparts. We show that CD57+ PD-1 TFH cells exhibit a cytotoxic transcription signature characterised by expression of CRTAM, a recently described master regulator of murine cytotoxic CD4+ T cells, but only a weak cytotoxic phenotype. By contrast, circulating CD57+ PD-1 CD4+ T cells are rare, but exhibit a prominent cytotoxic phenotype. We present evidence consistent with a model in which STAT3 regulates this cytotoxic signature, but cells that express high levels of PD-1, such as CD57+ TFH, become refractory to STAT3.

## Results

### CD57 expression by PD-1+ CD4+ T cells in tonsil and blood

**Figure 1:**
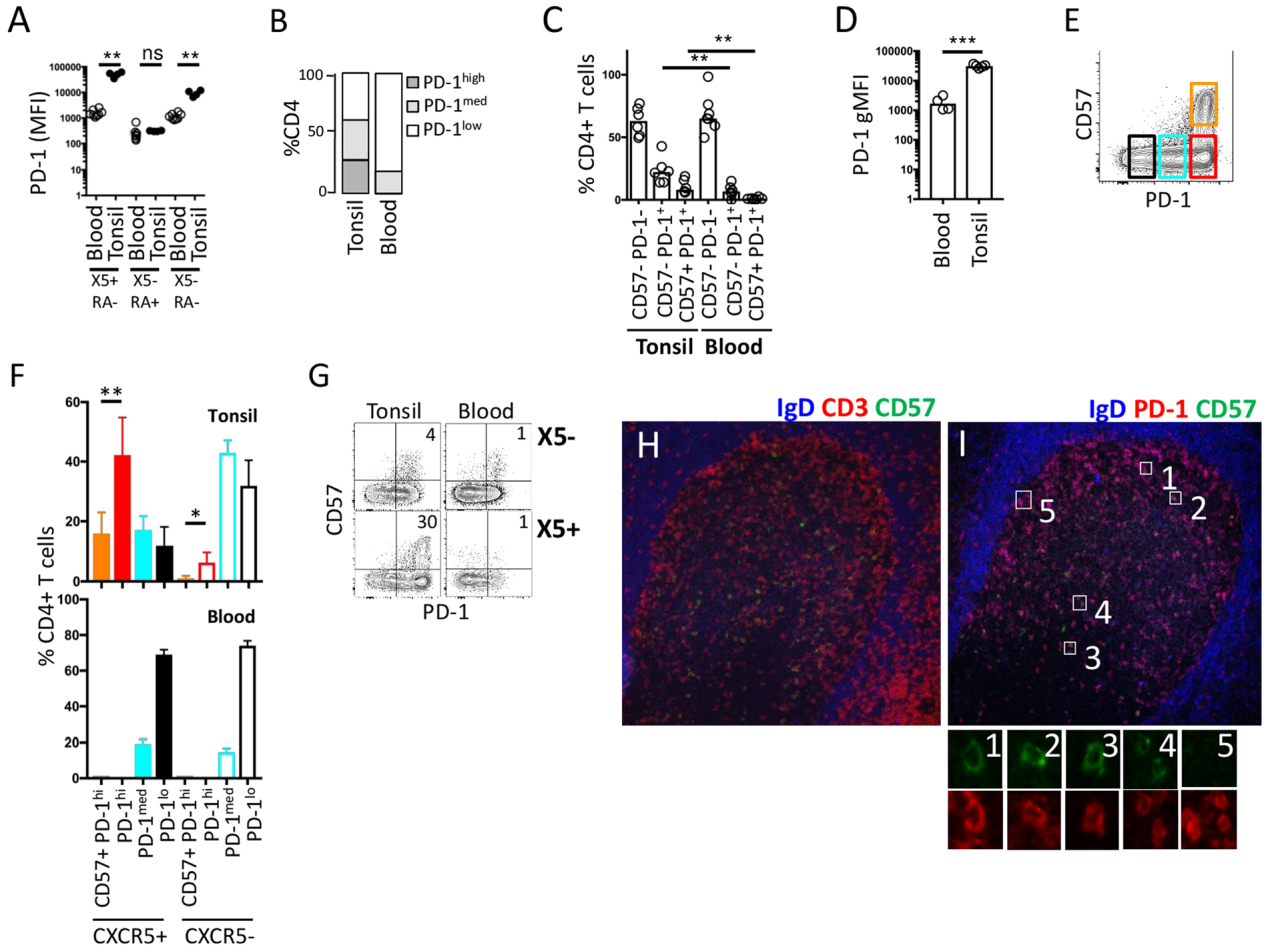
Distribution of CD57+ CD4+ T cells in blood and tonsil. (**A**) Summary of expression (mean fluorescence) of PD-1 by CD4+ T cell subsets defined as TFH (CXCR5+, X5+; CD45RA−; RA−), conventional memory (CXCR5−, X5−; CD45RA−, RA−), and naïve (CXCR5−, X5−; CD45RA+, RA+) in blood (n = 10) and tonsil (n = 4). (**B**) Summary of relative abundance in PBMC (n = 10) and tonsil (n = 4) of CD4+ T cells defined according to PD-1 levels (low, medium and high), using mean data from part A. (**C**) Summary of flow cytometric analysis of single cell suspensions from tonsil (n = 6) and PBMC (n = 7), indicating the proportion of cells within the indicated subsets. (**D**) Summary of mean fluorescence levels of PD-1 on CD57+ CD4+ T cells from blood (n = 4) and tonsil. (n = 7) (**E)**. Representative flow cytometric analysis of four CD4+ T cell tonsil subsets determined by PD-1 and CD57 expression. (**F**) Summary of abundance of CD57 and PD-1 expression in blood and tonsil according to presence or absence of CXCR5. Tonsil, n = 4; blood, n = 4. (**G**) Representative flow cytometry of tonsil and blood suspensions for expression of PD-1 and CD57 in CXCR5+ (X5+) and CXCR5− (X5−) compartments. (**H,I**) Representative immunofluorescence images of CD57+ T cells in sections of tonsil, stained for CD57, CD3 and IgD (**F**) and IgD, PD-1 and CD57. Higher magnification of individual cells is shown below. *p < 0.05, **p < 0.01, ***p < 0.001 (Fig. 1A,C,D and F).

PD-1 is known to be expressed at high levels by CXCR5+ CD45RA− TFH cells in secondary lymphoid organs^27^. We examined PD-1 expression on both CXCR5+ and CXCR5− CD4+ T cell subsets in paired blood and tonsil samples and found an overall bias towards PD-1 expression in tonsil. Indeed, for each defined CD4+ T cell subset, PD-1 levels are higher in tonsil than blood (Figs 1A,B, S1A). CD57+ CD4+ T cells represent a small but significant proportion of GC TFH (CXCR5+) cells whereas CD57+ PD-1+ cells are rare in blood, accounting for approximately 1% of CD4+ T cells versus approximately 10% in tonsil (Fig. 1C). In both blood and tonsil, almost all CD57+ cells express high level PD-1, although consistent with other subsets, PD-1 expression is higher on TFH than CD57+ cells in blood (Figs 1D, S1B).

When tonsil CD4+ T cells are analysed by PD-1 and CD57 expression, four distinct memory populations can be identified: PD-1^lo^CD57^−^, PD1^+^CD57^−^, PD1^hi^CD57^−^, PD1^hi^CD57^+^ (Fig. 1E). The relative abundance of these subsets in blood and tonsil is shown in Fig. 1F. The bias to PD-1 expression is greatest for CXCR5+ CD4+ T cells in tonsil relative to CXCR5− cells memory cells and circulating memory cells (irrespective of their CXCR5 expression). Since CD57 is expressed by PD-1^hi^ cells, most CD57+ cells are found within the CXCR5+ tonsillar subset (Fig. 1G). We found that the level of CXCR5 expression is similar on PD-1^hi^ cells irrespective of CD57 expression (Fig. S1C). Most CD57+ CD4+ T cells are CCR7− irrespective of whether they are in blood or tonsil, and almost all CD57+ TFH cells are CCR7− (Fig. S1D). TFH are located in germinal centres, where they are found in the light zone (Fig. 1H,I)^6,12^.

In summary, CD57 is expressed predominantly by CCR7− CD4+ T cells in blood and tonsil, there is a bias to both PD-1 and CD57 expression in tonsil relative to blood, and in tonsil CD57+ cells are present in mainly in the CXCR5+ fraction, while in blood they are mainly present within the CXCR5− fraction. (Fig. 1G).

### CD57+ PD-1^hi^ CD4+ T cells exhibit attenuated cytokine production

**Figure 2:**
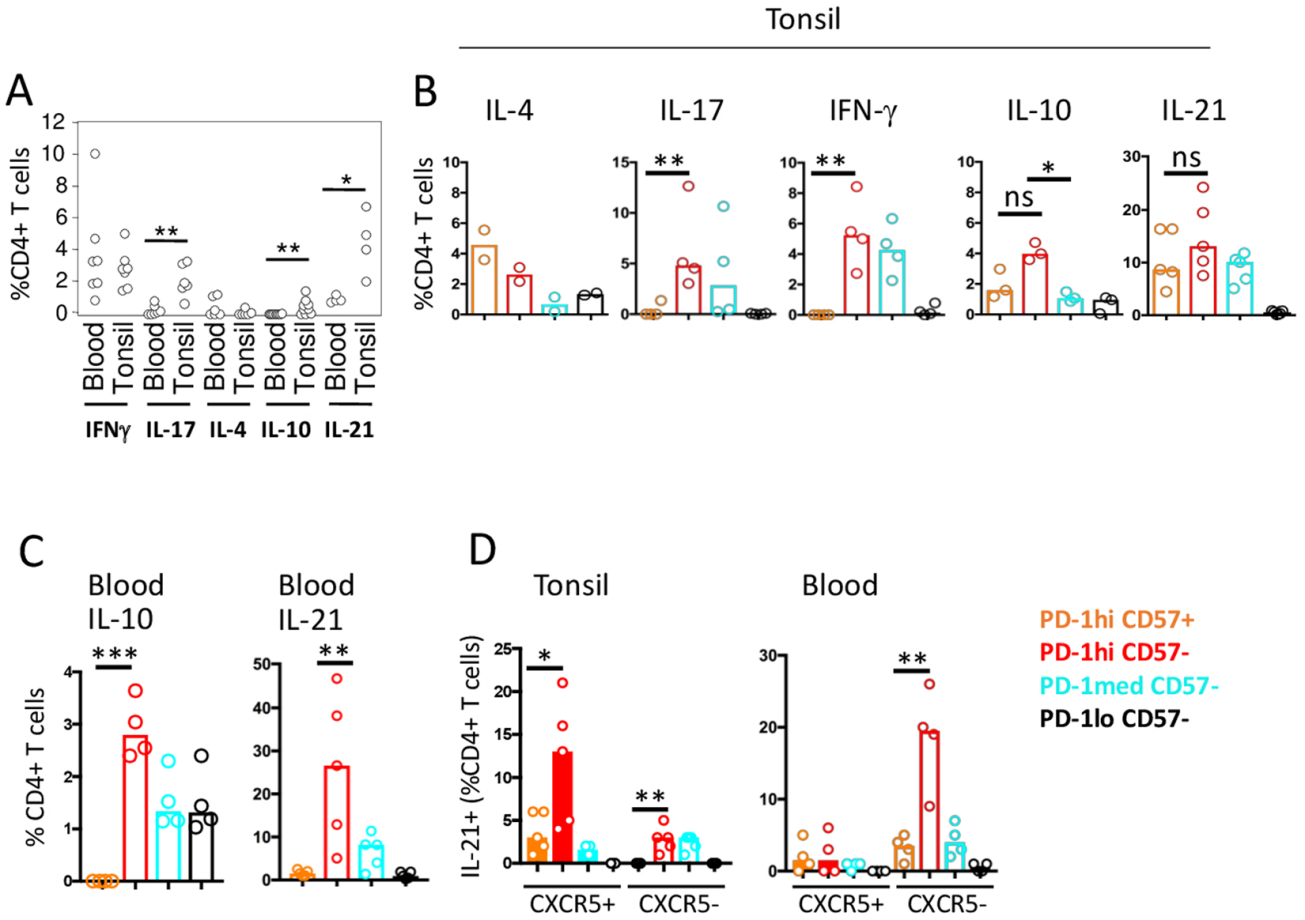
Cytokine production according to PD-1 and CD57 expression. (**A**) Summary of relative abundance of CD4+ T cells expressing the indicated cytokines in tonsil and blood, determined by intracellular staining and flow cytometry of paired samples from individual donors (n = 7, except for IL-21 where n = 4). (**B**) Summary of relative abundance of intracellular cytokines in purified CD4+ T cell subsets defined in Fig. 1H. Each symbol indicates a result from a different donor (IL-4, n = 2; IL-17, n = 4; IFN-γ, n = 4; IL-10, n = 3; IL-21, n = 5). (**C**) Summary of relative abundance of IL-21 (n = 5) and IL-10 (n = 4) positive cells in peripheral blood subsets. (**D**) Summary of abundance of IL-21 producing cells in each indicated CD4+ T cell subset from tonsil (n = 5) or blood (n = 4), analysed according to CXCR5 expression. *p < 0.05; **p < 0.01, ***p < 0.001.

It remains uncertain whether there is a functional difference between CD57+ and CD57− PD-1^hi^ cells. Comparison of blood and tonsil CD4+ T cells isolated from the same donors for production of signature T helper cytokines revealed a significant bias towards production of STAT3-dependent cytokines IL-17, IL-10, and IL-21 in tonsil (Fig. 2A). We investigated this further to determine which tonsil subsets account for this bias, by examining cytokine expression by CD4+ T cell subsets defined by CD57+ and PD-1. All tested cytokines were detected in the CD57− PD-1^hi^ subset but there was significant attenuation of IL-17 and IFN-γ production in CD57+ PD-1^hi^ CD4+ T cells relative to their CD57− counterparts (Fig. 2B). IL-21 and IL-10 positive cells remained detectable in the CD57+ compartment although they were fewer compared to their CD57− PD-1^hi^ CD4+ T counterparts. Interestingly, CD57+ PD-1^hi^CD4+ T cells were found to be relatively enriched for IL-4, although the proportion of IL-4+ cells was small. While PD-1^hi^ CD4+ T cells are rare in peripheral blood, we observed similar patterns of cytokine expression, but with a significant enrichment for IL-21 and IL-10 production in CD57− PD-1^hi^ cells compared to CD57+ PD-1^hi^ CD4+ T cells (Fig. 2C). Finally, we analysed IL-21 production in CXCR5+ and CXCR5− cells from blood and tonsil (Fig. 2D). In tonsil, IL-21 is predominantly produced by CXCR5+ CD4+ T cells, and we identified a significant bias towards cytokine production by CD57− PD-1^hi^ cells relative to CD57+ PD-1^hi^ cells. The same pattern was seen in CXCR5− tonsillar CD4+ T cells. Interestingly in blood, there is a similar bias to IL-21 production by CD57− PD-1^hi^ cells mainly in CXCR5− CD4+ T cells (Fig. 2D).

### CD57+ CD4+ PD-1^high^ T cells are not T follicular regulatory cells (TFR)

**Figure 3:**
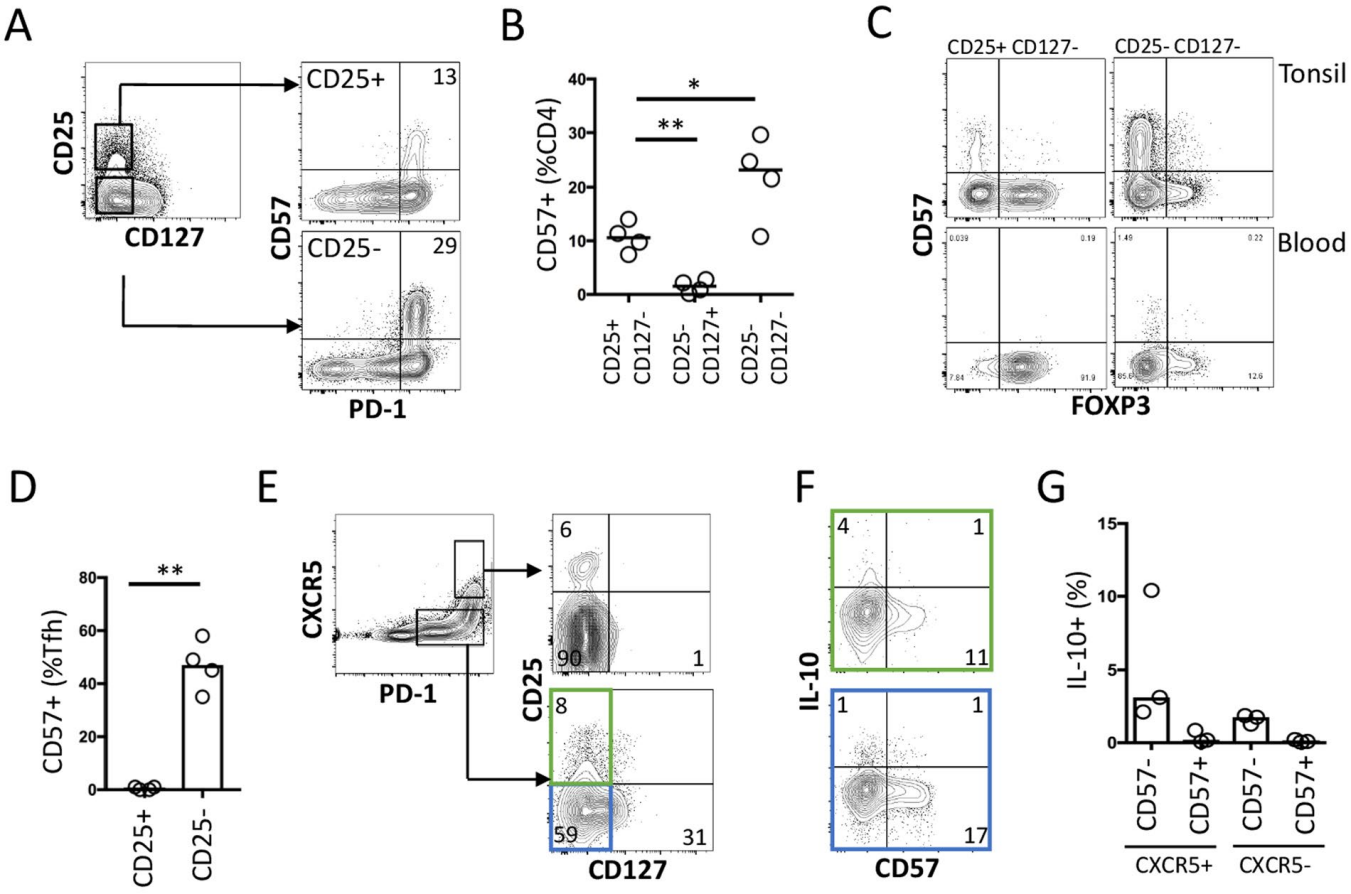
CD57+ CD4+ T cells are not enriched with TFR cells. (**A**) Flow cytometric analysis of either CD25+ CD127− or CD25− CD127− CD4+ T cells for expression of CD57 and PD-1. (**B**) Summary of analysis in (**A**) (n = 4). (**C**) Representative analysis of CD4+ CD25+ CD127− T cells obtained from either blood or tonsil for FOXP3 and CD57 expression according to CD25+ or CD25− co-expression. (**D**) Summary of CD57+ cells within CD25+ or CD25− TFH (CXCR5^hi^ PD-1^hi^) (n = 4). (**E**) Analysis of TFH and non-TFH PD-1+ cells for expression of CD25 and CD127. (**F,G**) Analysis and summary of proportion of IL-10 expression in CD57+ cells after gating on indicated regions in (**E**) (n = 3). *p < 0.05; **p < 0.01.

Since CD57+ TFH showed reduced capacity to make cytokines, but retained the capacity to make IL-10, we investigated whether CD57+ TFH are in fact TFR cells. This does not appear to be the case, as CD25+ CD127^lo^ cells were predominantly CD57− (Fig. 3A,B). CD127− cells in tonsil were enriched for PD-1^hi^ cells (Fig. 3A) and CD57+ but were FOXP3− (Fig. 3C). Almost all CD57+ CXCR5+ CD4+ T cells from tonsil are CD25− (Fig. 3D). Finally, we analysed TFH subsets for IL-10 production. TFH and TFR were gated according to PD-1, CXCR5, CD127 and CD25. In both CXCR5+ and CXCR5− subsets, most IL-10 production was by CD57− cells (Fig. 3F,G).

### The action of STAT3 on CD57+ PD-1hi CD4+ T cell abundance

**Figure 4:**
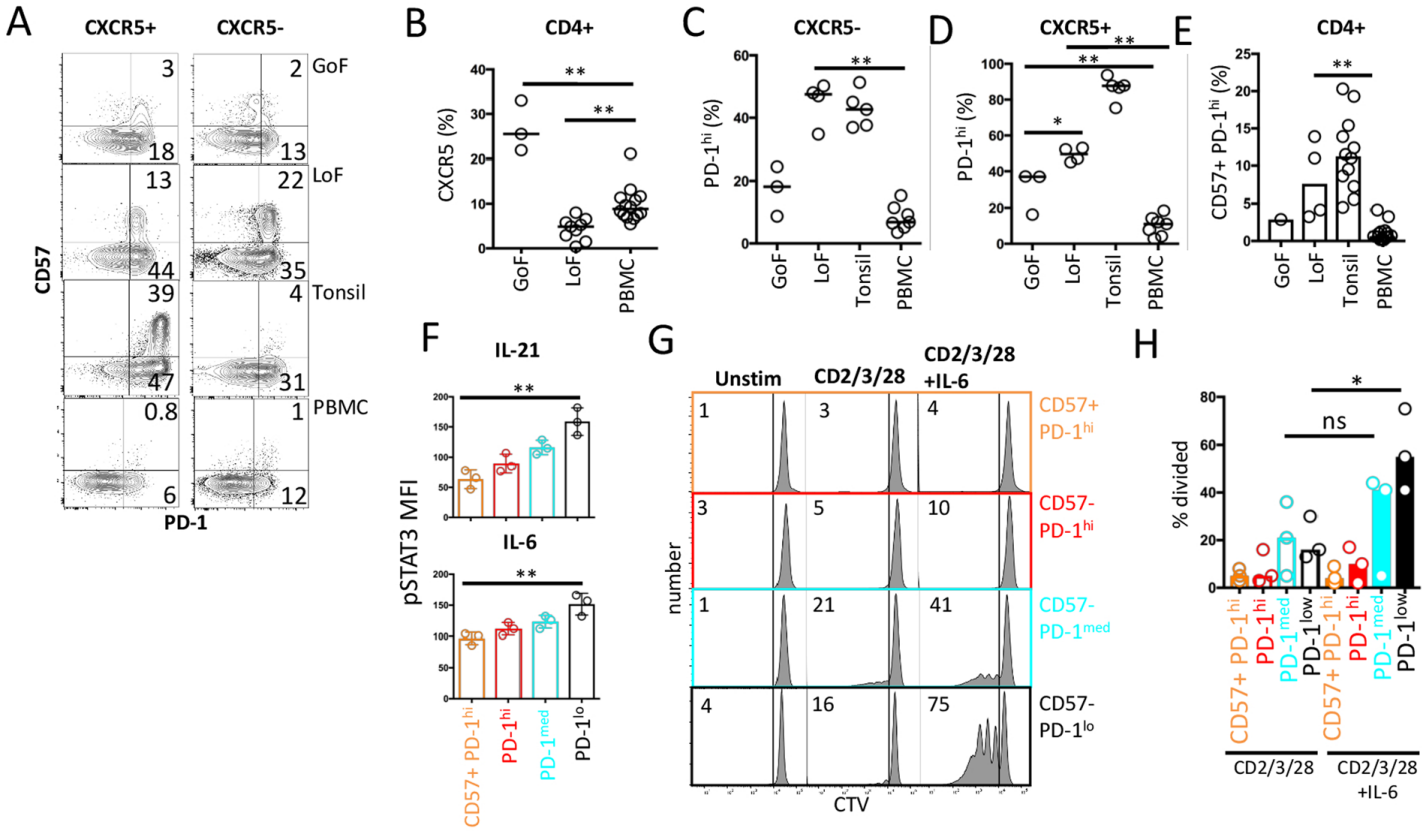
STAT3 modifies abundance of CD57+ CD4+ T cells. (**A**) Representative flow cytometric analysis of abundance of CD57+ cells in CXCR5+ and CXCR5− compartments in blood from patients with *STAT3* loss-of-function (LoF), gain-of-function (GoF) mutations, or from PBMC of normal donors, or tonsil. (**B**) Summary of TFH cells (CXCR5+ CD45RA−) cells from *STAT3* GoF (n = 3), STAT3 LoF (n = 9) or normal donors (n = 15). (**C,D)** Summary of abundance of PD-1^hi^ cells (as a percentage of total CD4+ T cells) in CXCR5− (**C**) and CXCR5+ (**D**) compartments in PBMCs from STAT3 GoF (n = 3), LoF (n = 4), healthy donors (n = 7) or tonsil (n = 5). (**E**) Summary of PD-1+ CD57+ subset of CD4+ T cells in each indicated group, *STAT3* GoF (n = 1), LoF (n = 4), healthy donors (n = 14) or tonsil (n = 12). (**F**) Summary of phosphorylated STAT3 expression after stimulation of each PD-1 and CD57+ subset with either IL-6, IL-21 (n = 3). (**G**). Representative flow cytometric analysis of *ex vivo* proliferation on day 4 of each purified CD4+ T cell subset (as labelled) after stimulation with CD2/3/28 in the presence or absence of IL-6. (**H**) Summary of proliferation of CD4+ T cell subsets (n = 3). *p < 0.05; **p < 0.01.

In the absence of any evidence that CD57+ PD-1^hi^ TFH cells are TFR we sought other clues about their function based on their ontogeny. Previous evidence has shown that the abundance of CXCR5+ CD4+ T (cTFH) cells is partially dependent on STAT3, as revealed by analysis of CD4+ T cells from individuals with dominant negative heterozygous missense mutations in *STAT3*^28–30^. In patients with either GoF or LoF *STAT3* mutations, lymphocytes are expected to be under chronic antigenic stimulation (by either self- or foreign antigen)^31–34^. Examination of CD4+ T cells from patients with *STAT3* GoF mutations revealed an increase in CXCR5+ CD4+ T cells (cTFH) in blood (Fig. 4A,B). Moreover, relative to normal blood samples, blood samples from patients with *STAT3* GoF expressed an increase in the proportion of PD-1^hi^ cells, but not as marked as had been observed in blood samples from patients with *STAT3* LoF (Fig. 4A,C–E). However, in contrast to blood samples of patients with *STAT3* LoF, there was no expansion of CD57+ cells in blood samples of patients with *STAT3* GoF (Fig. 4A,E).

While *STAT3* LoF causes a deficiency in cTFH, there was an increase in PD-1^hi^ CD4+ T cells in those samples (Fig. 4C,D). Indeed, when PD-1^hi^ cells were expressed as a proportion of each parent population, STAT3 deficiency results in a significant increase of PD-1^hi^ cells in both CXCR5+ and CXCR5− populations compared to normal donors (Fig. 4C–E). The abundance of PD-1^hi^ cells among CXCR5− CD4+ T cells was similar in normal tonsils and blood of patients with *STAT3* LoF (Fig. 4C,D). We observed that *STAT3* LoF also resulted in increased PD-1 expression per cell in circulating CD4+ T cells^35^. As observed in normal tonsil, this increase in peripheral blood CD57+ CD4+ T cells in *STAT3* LoF patients was found to be predominantly within the PD-1^hi^ population; however, these were mostly CXCR5− CD4+ T cells (Fig. 4A). The surface phenotype described so far suggests that *STAT3* LoF resulted in circulating CXCR5− cells which adopt a phenotype (CD57+ PD-1^hi^), which is similar to that observed in CXCR5+ cells (TFH) in tonsil (Fig. 4C–E).

### STAT3 responsiveness is impaired in PD-1^high^ CD4 cells

With this clue that *STAT3* LoF confers enrichment of CD57 PD-1^hi^ phenotype, similar to that observed in tonsils, we sorted normal tonsil CD4+ T cells into the four populations defined by PD-1 and CD57 expression (Fig. 1E), and examined STAT3 phosphorylation in each subset. We observed an inverse relation between PD-1 expression and pSTAT3 induction in response to IL-21 and IL-6, with virtually no pSTAT3 expression in PD-1^high^ CD4+ cells regardless of their CD57+ expression (Fig. 4F). We also tested whether this translated into a differential T cell proliferation response. Again, each T cell subset was sorted from tonsils, labelled with CTV and analysed for proliferation after stimulation with CD2/3/28 in the presence or absence of the STAT3-dependent cytokine, IL-6. We observed that IL-6 augmented the response in PDi-1^lo^ and PD-1^med^ subsets, but not PD-1^hi^ cells, again, irrespective of their CD57 expression (Fig. 4G,H). Similarly, activation of purified T cells with anti-CD2/3/28 and either IL-6 or IL-21 resulted in induction of IL-10 by PD-1^int^ cells, whereas we found no cytokine induction in PD-1^hi^ subsets (Fig. S2). Taken together, these findings indicate PD-1^hi^ phenotype regardless of the CD57 expression, marks cells that are unresponsive to STAT3.

### CD57+ CD4+ T cells exhibit a cytotoxicity gene expression signature

**Figure 5:**
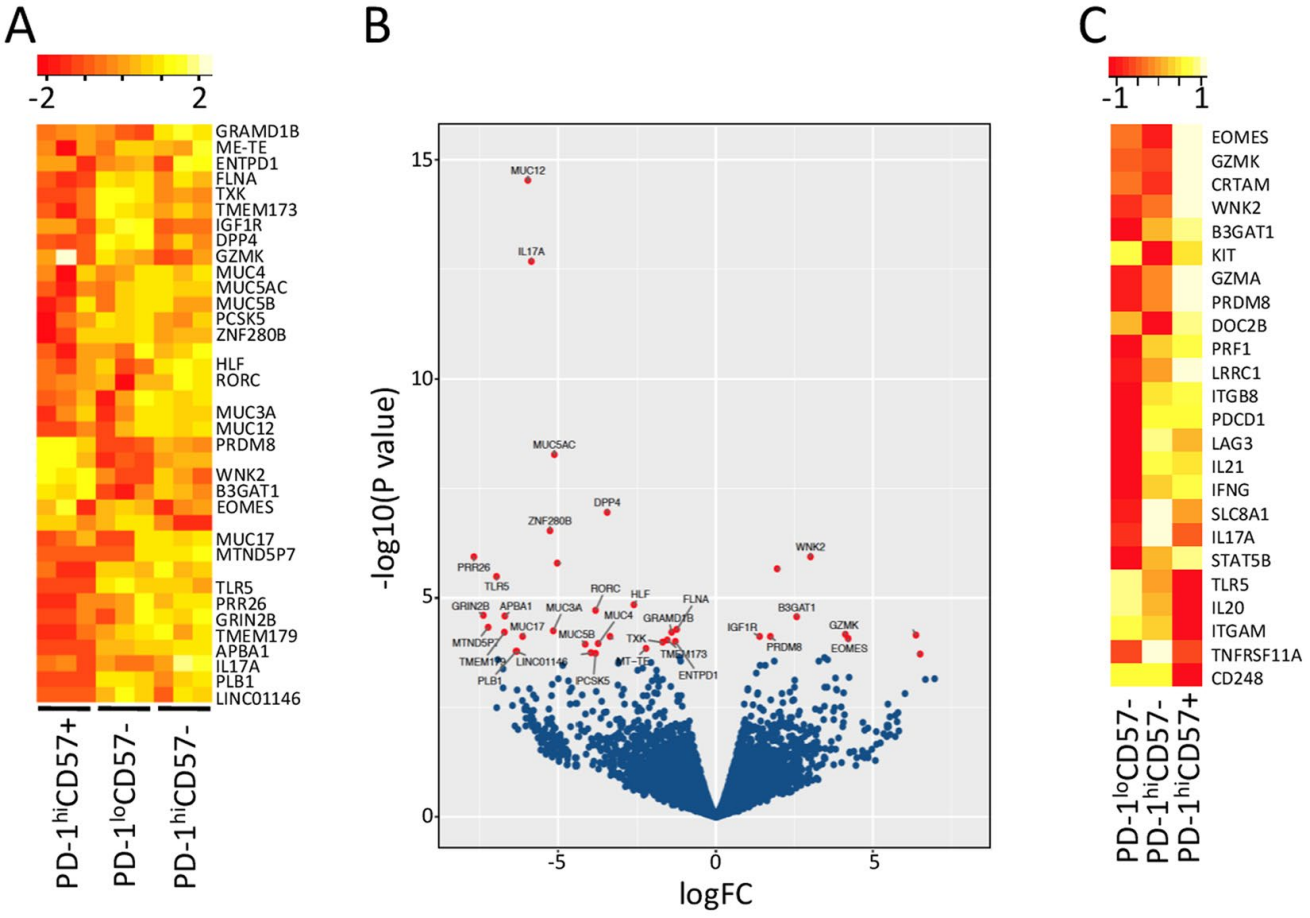
Analysis of global gene expression. (**A**) Relative gene expression (determined by RNA-Seq) in purified PD-1^hi^ CD57+, PD-1^hi^ CD57− and PD-1^lo^ CD57− cells (n = 3 biological replicates per subset). (**B**) Volcano plot showing log fold change on x-axis and Log P Value on y-axis in the PD-1^hi^ CD57+ versus PD-1^hi^ CD57− cells comparison. The tonsil 2 samples were excluded from this analysis. Genes reaching statistical significance (FDR < 0.1) are highlighted (red). (**C**) Heatmap of average expression values (excluding T2) for the 3 conditions PD-1^hi^ CD57+ vs PD-1^lo^ CD57−, (column 2) and PD-1^hi^ CD57− vs PD-1^lo^ CD57− (column 1) comparisons (n = 2 biological replicates).

Our findings described so far suggested that PD-1^hi^ exhibit relative proliferative incompetence regardless of their CD57+ expression. Moreover, CD57+ PD-1^hi^ TFH produce less cytokines compared to their CD57− counterparts suggesting they might be merely exhausted T cells. To test whether CD57+ cells might have a positive function, we compared gene expression by CD57+ and CD57− TFH subsets by RNA-Seq (Fig. 5A). Despite the considerable similarity of gene expression patterns in CD57+ and CD57− PD-1^hi^ CD4+ T cells in the small sample tested, we identified some important differences. There was an increased expression of *EOMES*, *WNK2, CRTAM, GZMK and PRDM8* in the CD57+ subset, while *TLR5* was expressed at lower levels in the CD57+ subset (Fig. 5B). A heatmap of the average expression values shows the trend towards increased expression of *EOMES, WNK2, CRTAM, GZMA, GZMK* and *PRDM8* in the CD57+ cells and the trend towards lower expression of *TNFRSF11A, CD248, ITGAM, TLR5* in the CD57+ cells compared to CD57− CD4+ T cells (Fig. 5C).

**Figure 6:**
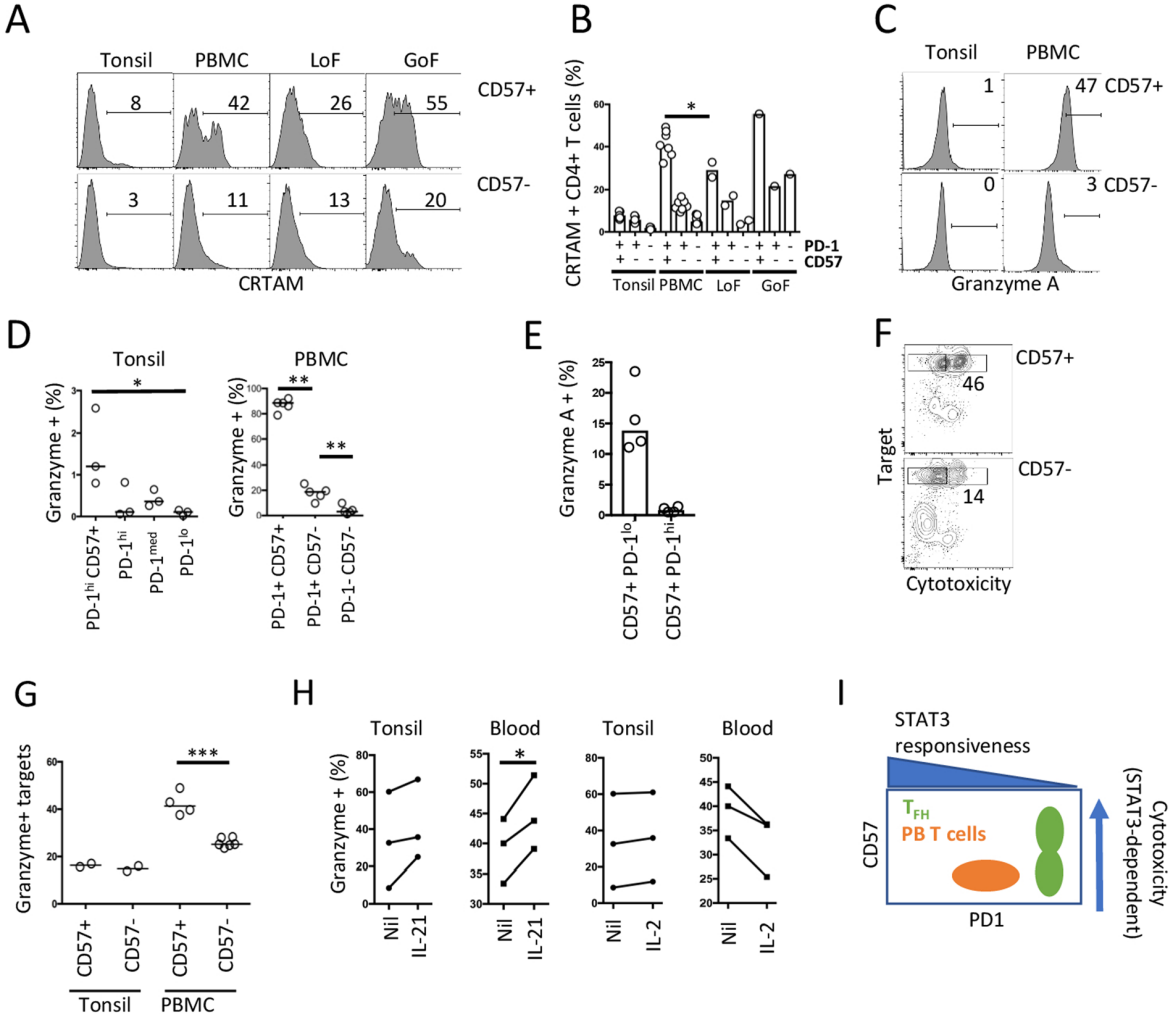
Cytotoxicity phenotype in CD57+ CD4+ T cells. (**A**) Representative flow cytometric analysis of CRTAM expression in tonsil (n = 3) and blood (n = 7) CD4+ T cells from healthy donors, or patients with STAT3 LoF (n = 2) or GoF (n = 1), gated on PD-1+ CD57− or PD-1+ CD57+, with summary results (**B**). (**C**) Representative analysis of intracellular granzyme A expression in tonsil and blood gated on PD-1^hi^ CD57− or PD-1^hi^ CD57+. (**D**) Summary of granzyme positive cells in tonsil (n = 3) and PBMC (n = 5) in CD4+ T cell subsets defined by PD-1 and CD57. (**E**) Summary of percentage of granzyme positive cells in PD-1hi and PD-1medium CD57+ CD4+ T cells. (**F,G)** Representative flow cytometric analysis of cytotoxicity of target cells co-cultured with CD57+ or CD57− CD4+ T cells, expressed graphically in E, in tonsil (n = 2) and PBMC (n = 4), summary in **G**. (**H**) Summary of granzyme A induction on day 2 by PBMC (n = 3) and tonsil (n = 3) cells in response to IL-21 or IL-2. Paired results from each donor are connected. (**I**) Model relating CD57 and PD-1 expression to STAT3 responsiveness. *p < 0.05; **p < 0.01, ***p < 0.001.

CRTAM (MHC class-I restricted T cell associated molecule) has been reported recently as a master regulator of CD4+ cytotoxic T cells in mice^36^. Having obtained this clue to positive function for CD57+ PD-1^hi^ TFH cells by transcriptional analysis, we proceeded to analyse CD57+ and CD57− cells for a cytotoxic phenotype. In tonsil, we found that, the proportion of CRTAM+ cells in the CD57+ population was about twice that in CD57− cells in tonsil (Fig. 6A,B), although overall, the cytotoxic phenotype of CD57+ PD-1^hi^ tonsil T cells was weak. By contrast, analysis of circulating CD57+ CD4+ T cells showed substantial CRTAM expression, with approximately 4-fold more CRTAM+ cells in the CD57 compartment, than in the PD-1^hi^ CD57− compartment (Fig. 6A,B).

We also examined CD57+ and CD57− cells for granzyme expression. Consistent with gene expression, we observed a small but significant increase in granzyme A+ cells within the CD57+ compartment in tonsil, but a much more substantial proportion in circulating CD57+ cells (Fig. 6C,D). On analysis of all four tonsil subsets (defined by PD-1 and CD57−), most granzyme positive cells were located within the CD57+ PD-1^hi^ population (Fig. 6D). Of note, there is a rare population of CD57+ PD-1^lo^ cells in the tonsil, of which are higher proportion express granzyme when compared with more abundant CD57+ PD-1^hi^ cells (Fig. 6E). Similarly, in blood, where most granzyme A+ cells were CD57+, overall PD-1 expression levels are lower than in tonsil (Figs 1A and 6C,D). We tested functional cytotoxicity. Purified CD57+ and CD57− PD-1^hi^ CD4+ T cells were co-cultured with B cell targets, and we observed a consistent increase in cytotoxicity mediated by CD57+ cells in blood, but not tonsil (Fig. 6F,G).

### STAT3 regulates CD4+ T cell cytotoxicity independently of exhaustion

As reported above, PD-1^hi^ T cells from tonsil produce less cytokines and proliferate poorly, suggesting they might be exhausted cells. Moreover, in patients with either GoF or LoF *STAT3* mutations, lymphocytes are expected to be under chronic antigenic stimulation (by either self- or foreign antigen), we observed increased numbers of circulating PD-1^hi^ CD4+ T cells. However, more CD4+ T cells from patients with *STAT3* LoF adopted CD57+ PD-1^hi^ phenotype and we have shown above that CD57 CD4+ T cells have cytotoxic phenotype. We were therefore interested to determine whether STAT3 regulates this cytotoxic potential of CD4+ T cells or only contributes to the exhaustion phenotype. We examined CD4+ T cells from patients with either *STAT3* GoF or LoF. Despite the expansion of CD57+ cells conferred by STAT3 Lof, we observed a smaller proportion of CRTAM+ cells in this compartment compared with normal PBMCs (Fig. 6B), whereas there is a suggestion of the opposite phenotype with STAT3 GoF patient (Fig. 6A–C). To confirm the importance of STAT3 signaling for cytotoxicity, we stimulated tonsil and PBMC CD4+ T cells with either IL-21 or IL-2. We observed that IL-21 promoted granzyme expression, to a greater extent in blood than tonsil. We observed a similar effect of IL-6 (Suppl Fig. 3), while IL-2 had the opposite effect (Fig. 6H). This action of IL-21 is not accounted for simply by differences in IL-21 receptor expression (Supp. Fig. 3). Taken together, these findings indicate that STAT3 regulates cytotoxicity, but PD-1^hi^ TFH cells are refractory to this action.

## Discussion

We set out to determine whether CD57+ PD-1^hi^ TFH cells have a distinct function and conclude that they exhibit a defect in signature cytokine production relative to CD57− CD4+ T cells that express similar level of PD-1. In addition, we show that CD57+ TFH fail to respond to cytokines that signal through STAT3, and proliferate poorly, but these characteristics are similar in both PD-1^hi^ TFH cells irrespective of CD57. CD57+ CD4+ TFH cells are distinguished from their CD57− PD-1^hi^ counterparts by the expression of a cytotoxicity signature, but this translates into only a weak cytotoxic phenotype. These observations led us to identify several more general conclusions regarding CD4+ T cell cytotoxicity. First, CD57 expression identifies CD4+ T cells with cytotoxic activity, based on transcriptional signature and phenotype. Second, we show that cytotoxicity is promoted by STAT3 signalling. Third, we show that high levels of PD-1 expression flags cells that are unresponsive to STAT3. Consequently, while CD57 identifies CD4+ T cells with cytotoxic potential, this effect is offset when they adopt high level PD-1 expression within secondary lymphoid organs. Thus, a large fraction of the rare circulating CD57+ CD4+ T cells have a cytotoxic phenotype. By contrast, CD57+ CD4+ TFH cells, which are abundant in germinal centres, are universally PD-1^hi^ and exhibit a weak cytotoxic phenotype, despite their cytotoxicity transcriptional signature.

This findings lead us to propose a model in which there exists a trade-off between acquisition of the cytotoxic phenotype, and abundance of cells that can adopt this phenotype (Fig. 6I). According to this model, STAT3 unresponsiveness, marked by high expression of PD-1, also results in refractoriness to adoption of a cytotoxic phenotype, and this appears to be the usual phenotype of CD57+ TFH in germinal centres. By contrast, in blood, CD57+ expression occurs on cells that are PD-1+, but express PD-1 at lower levels than observed on TFH cells, and CD57+ cells in blood adopt a cytotoxic phenotype. Thus, while CD57 identifies CD4+ T cells with cytotoxic potential it is not the abundance of CD57+ CD4+ T cells but their PD-1 expression that predicts the proportion that adopt the cytotoxic phenotype.

STAT3 exhibits a complex regulatory action on TFH differentiation. Previous work has shown that dominant negative *STAT3* mutations result in a reduction in CXCR5+ CD4+ T cells in blood^28^. We confirmed those findings, and in addition, demonstrate that the converse is true for *STAT3* GoF, which confers a relative increase in the proportion of circulating CXCR5+ CD4+ T cells. Whereas *STAT3* LoF is associated with increased PD-1 expression by circulating CD4+ T cells, plus an increase in the proportion of PD-1^hi^ cells that are CD57+, these changes are not observed with *STAT3* GoF. Although the CD57+ CD4+ T cell fraction is increased in the blood in patients with *STAT3* LoF, this is not associated with enhanced cytotoxicity, as judged by CRTAM expression. CD4+ T cells from tonsil also exhibit an overall bias towards increased PD-1 expression, based on comparison of each identifiable T cell subset in blood and tonsil from the same individuals. Tonsil CD4+ T cells appear to be less responsive to STAT3, as judged by STAT3 phosphorylation, response to cytokines that signal via STAT3, and proliferation in response to IL-6. We infer from these observations that while STAT3 is necessary for adoption of the CXCR5+ phenotype, differentiation of CD4+ T cells to a PD-1^hi^ phenotype is associated with STAT3 unresponsiveness.

We identified expression of CRTAM as an important difference between CD57+ and CD57− CD4+ T cells. CRTAM was identified in mouse CD4+ T cells as a master regulator of the CD4+ cytotoxic T cell subset^36^. We showed relative increase in CRTAM expression in CD57+ PD-1^hi^ cells relative to PD-1^hi^ CD57− cells flagged a potential cytotoxic signature in this germinal centre subset. Further analysis, however, suggested that relatively few germinal centre cells express either CRTAM protein or granzyme. By contrast, these proteins are abundant in blood CD4+ CD57+ T cells. Our findings are consistent with CRTAM regulating cytotoxicity in the CD4+ T cell compartment in mice and humans.

In addition to cytotoxicity, we observed that cytokine production varies qualitatively according to PD-1 and CD57 expression. In the tonsil, we defined four CD4+ T cell subsets using these markers, and showed that IFN-γ and IL-17 expression and, to a lesser degree, IL-21 and IL-10 is higher as a proportion of the cells in the PD-1^hi^ subset, but in the CD57+ fraction of PD-1^hi^ cells, cytokine production is attenuated. A significant bias towards IL-21 and IL-10 is observed in rare PD-1^hi^ cells in blood, and appears to be independent of CXCR5 expression. Interestingly, comparison of matched cell subsets from individual donors shows a bias towards production of STAT3-dependent cytokines IL-21, IL-17 and IL-10 in tonsil relative to blood. In view of the cellular subset analysis, this bias appears to be accounted for by the greater abundance of PD-1^hi^ cells in the tonsil.

These findings suggest overall similarities between blood and tonsil PD-1+ CD4+ T cells. In the circulating CD4+ T cell compartment, the CD57+ subset of PD-1+ CD4+ T cells is enriched with cells exhibiting a cytotoxic phenotype. These cells are rare in the peripheral blood, but a majority express granzyme and CRTAM, and are also cytotoxic by functional analysis. We show directly, that IL-21, which signals through STAT3, promotes granzyme expression, and consistent with this observation, a higher than normal proportion of CD4+ T cells are cytotoxic (as shown by higher number of cells expressing CRTAM) in blood in the presence of STAT3 GoF. Thus, in both blood and tonsil, CD57 expression marks cells with cytotoxic phenotype, and yet in tonsil, where CD57+ CD4+ T cells are most abundant, cytotoxicity is weak. One inference from these observations is that there is normally a greater constraint on CD57+ cell formation in blood than tonsil, and a greater constraint on acquisition of a cytotoxic phenotype by CD57+ CD4+ T cells in tonsil relative to their circulating counterparts.

An explanation for the acquisition of STAT3 unresponsiveness in GC will require further investigation. Expression of CD57 and adoption of cytotoxic potential could reflect chronic antigenic stimulation within GCs. Indeed, adoption of cytotoxicity within the CD57+ T cell compartment in blood has also been observed in states of chronic antigenic stimulation^37^. Similarly, CD57 expression is observed in the CD8 compartment under similar circumstances. Previous investigations have suggested that CD57+ and CD57− cells are similar with regard to capacity to provide B cell help^12,13^. Consistent with this similarity in B cell help, the difference between CD57+ and CD57− cells, at least by global transcription, is relatively small. Furthermore, both CD57+ and CD57− cells express comparable levels of BCL6^38^. Nevertheless, CD57− PD-1^hi^ cells produce abundant IL-21 and IL-10, a phenotype consistent with greater potential for T cell help than PD-1^lo^ CD4+ T cells.

The function of cytotoxicity within the GC is open to speculation. While there are conditions that operate within the GC that appear to result in a bias away from cytotoxicity, there remains a small and measurable cytotoxic subset within the TFH compartment. Recent evidence indicates the cytotoxic CD8+ T cells exist in the GC, also consistent with cytotoxicity as an immunoregulatory mechanism in the GC^39^. If adoption of cytotoxic potential by terminally differentiated TFH does regulate B cell responses, our findings suggest that help (IL-21 and IL-10 from CD57− PD-1^hi^ CD4+ TFH) adoption of cytotoxicity (by CD57+ PD-1hi CD4+ TFH) is on a continuum, regulated by intensity of STAT3 signalling. This contention is supported indirectly by the observation of the paradoxical coincidence of antibody deficiency and autoimmunity observed with some forms of primary immune deficiency, including *STAT3* GoF^32,34^. In addition, our observations might provide in new opportunities for tuning immunity by altering modulating help versus cytotoxicity through alteration of STAT3 signalling. Finally, we note that interruption of ligation of PD-1 enhances anti-tumour responses, and alter the natural history of several types of cancer, although the effect is sometimes limited in duration^40,41^. In the light of our findings, monitoring CRTAM expression within CD57+ and PD-1^hi^ CD4+ T cells might be a useful strategy for monitoring immunotherapy.

## Materials and Methods

### Patients and samples

Paired tonsils and peripheral blood mononuclear cells (PBMCs) samples were obtained from patients undergoing routine tonsillectomy and PBMCs alone from healthy controls and patients’ groups as indicated. Biological samples were obtained after obtaining written informed consent from donors. All procedures were performed in accordance with the relevant guidelines and regulations and were approved by Australian Capital Territory Health Human Research Ethics Committee and Australian National University Human Research Ethics Committee.

### Antibodies and reagents

The following antibodies were used for flowcytometry: CD3 (HIT3a, Becton-Dickinson (BD)), CD4 (SK3, BD), CD45RA (HI100, Biolegend), CXCR5 (TG2/CXCR5,Biolegend and RF8B2,BD), CCR7 (3D12, BD), PD-1 (NAT), CD25 (2A3, BD), CD127 (A7R34, eBioscience), CRTAM(R&D), Granzyme A (GB11,BD), Foxp3 (259D/C7, BD), CD57(HNK-1,BD), IL-17 (SCPL1362, BD), IL-10 (JES3-19F1,BD), IFN-g (25723.11,BD), IL-4 (7A3-3,BD), IL-21 (3A3-N2.1, BD), STAT3(4/P-STAT3,BD), and cell trace violet (CTV,Life Technologies). Data were acquired on FACSCaliber or FACSCanto II and data were then analyzed with FlowJo, version (8.7) (Treestar).

### STAT phosphorylation

PBMCs were rested for 2 hours at 37 °C (5% CO_2_), left unstimulated or stimulated with indicated cytokine for 20 minutes then processed according to the manufacturer’s protocol (BD). The following were used: IL-6 (100 ng/ml, MiltenyiBiotec), IL-21 (100 ng/ml, MiltenyiBiotec).

### Cytotoxicity assay

PanToxiLux kit was used according to manufacturer’s instructions (OncoImmunin). Indicated cell populations were incubated with targets cells (labelled TFL4) at various ratios. Targets were analysed at 1 hour by flow cytometry for the presence of intracellular granzyme.

### T cell proliferation assay

Indicated cell populations were labeled with Cell Trace Violet (CTV). For T cell proliferation, cells were stimulated with T cell activation beads (CD2/3/28)+/− IL-6 (100ng/ml, MiltenyiBiotec) as indicated for 5 days.

### Cytokine detection

1 × 10^6^/ml cellswere stimulatedwith leukocyte activation cocktail (BD) for 4 hours, cells were processed using fixation/permeabilisation kit (Becton Dickinson) following manufacture’s protocol.

### Cytokine induction assays

CD4+ T cells (FACS sorted on CD3+ CD4+ CD45RO−) or FACS sorted tonsil CD4+ T cells were cultured with anti-CD2/CD3/CD28 and either IL-6 (100 ng/ml, MiltenyiBiotec) or IL-21 (100 ng/ml, MiltenyiBiotec), for 2 days.

### Immunofluorescence

Frozen sections of human tonsil (6 μm) were fixed (cold acetone for 10 min) and blocked (3% BSA). Sections were then stained using anti-CD3 (LN10, Dako) or anti-PD-1 (EH12.2H7, Biolegend) overnight at RT, followed by anti-mouse IgG Alexa Fluor 594 (Invitrogen) for 30 min at RT. The slides were then incubated with anti-CD57 FITC (HNK-1,BD) and anti-IgD BV421 (IA6-2, Biolegend) for 30 min at RT. Stained sections were mounted using Vectashield mounting media (Vector Laboratories, #H-1000). Images were collected with an Olympus iX71 microscope with DP controller software (Olympus) using a 20× objective and analysed using ImageJ software.

### RNA-Seq

mRNA libraries for 9 samples were prepared at the Ramaciotti Centre for Genomics UNSW following the standard Illumina protocols. Sequencing was performed on the Illumina NextSeq 500 platform to produce 75 bp reads. There were 3 biological replicates (Tonsil 1, Tonsil 2 and Tonsil 3) for each of the three conditions: (1) PD-1^hi^CD57+, (2) PD-1^hi^CD57−, (3) PD-1^lo^CD57−. The generated reads were mapped to the Ensembl*Homo sapiens* genome (GRCh38) using Tophat2 (v 2.0.12)^42^ calling Bowtie2 (v 2.2.3)^43^. HTSeq-count (Python package HTSeq, python v 2.7.3)^44^ was used to generate counts of reads uniquely mapped to annotated genes using the GRCm38 annotation gtf file. We performed differential expression analysis using edgeR (v 3.12.0)^45^. An MDS plot indicated that samples from Tonsil 2 were outliers.Accordingly, we performed differential expression analysis without Tonsil 2 samples. Low count transcripts were excluded and only those genes with at least 1 count per million (cpm) in at least 3 samples were used for analysis. Differentially expressed genes were defined as those genes with a Benjamini-Hochberg corrected p value less than 0.1. We then mapped the results back onto the full data set using unsupervised clustering to produce heatmaps comparing expression levels of the genes of interest over all 9 samples.

### Statistical analysis

Data were analysed using Medcalc statistical software version 13.1.2 (MedCalc Software BVBA, Ostend, Belgium). Differences between the paired groups were analysed using either Mann Whitney test (independent sample) or Student’s t-test, and ANOVA for comparison of more than two groups.

## Electronic supplementary material

Supplementary information
