# Supplementary material for: STAT3 regulates cytotoxicity of human CD57+ CD4+ T cells in blood and lymphoid follicles
Source: Sci Rep. 2018 Feb 23;8:3529. doi: 10.1038/s41598-018-21389-8 (PMC5824848; doi:10.1038/s41598-018-21389-8)
Supplement: Supplementary file 1 — Supplementary information [file 41598_2018_21389_MOESM1_ESM.pdf]

STAT3 regulates cytotoxicity of human CD57+ CD4+ T cells in blood and lymphoid follicles

Jalila Alshekaili[1,2,3,4], Rochna Chand[1,2], Cindy Eunhee Lee[1,2], Susan Corley[5], Kristy Kwong[1, 2], Ilenia Papa[1], David A. Fulcher[1], Katrina L. Randall[1,3], Jennifer W. Leiding[6], Cindy S. Ma[7, 10], Marc R. Wilkins[5], Gulbu Uzel[8], Chris C. Goodnow[1,7, 9], Carola G. Vinuesa[1], Stuart G. Tangye[7,9], Matthew C. Cook[1,2,3].

[1] Department of Immunology and Infectious Disease, John Curtin School of Medical Research, Australian National University, ACT, 2601. Australia

[2] Translational Research Unit, Level 6 Building 10, Canberra Hospital, Woden, ACT, 2606. Australia

[3] Department of Immunology, Canberra Hospital, Woden, ACT, 2606. Australia

[4] Current address, Department of Microbiology and Immunology, Sultan Qaboos University Hospital, Oman

[5]Systems Biology Initiative, School of Biotechnology and Biomolecular Science, University of New South Wales, Sydney, Australia

[6]Division of Allergy, Immunology, and Rheumatology, Department of Pediatrics, University of South Florida, St Petersburg, Florida

[7]Immunology Division, Garvan Institute of Medical Research, 384 Victoria Street, Darlinghurst, NSW, 2010.

[8] Laboratory of Clinical Infectious Diseases, National Institute of Allergy and Infectious Diseases, National Institutes of Health, Bethesda, Maryland. USA

[9] St Vincent's Clinical School, Faculty of Medicine, University of New South Wales, Darlinghurst, NSW, 2010. Australia

Correspondence to

Matthew Cook, Department of Infection and Immunity, John Curtin School of Medical Research, Australian National University, ACT, 0200. Australia; T +61 2 6174 5586; F +61 2 6174 5847; E matthew.cook@anu.edu.au

Running title

Cytotoxic CD57+ CD4+ T cells

Supplementary figures legends

Fig. S1. PD-1 high cells have effector phenotype both in blood and tonsil

A. Representative flow cytometric analysis of level of PD-1 expression by different CD4+ T cells in blood and tonsils from the same donor according to expression of CXCR5 and CD45RA. Colours of the overlay histogram are derived from the quadrants in the dot plot. **B**. Flow cytometric analysis of expression of CXCR5 and PD-1 (right) in blood and tonsil gated on total CD4+ T cells (left panel) or according to CD57 status. **C**. Flow cytometric analysis of CXCR5 MFI on different PD-1 CD57 subsets in the tonsil, with summary plots from independent analyses. **D**. Flow cytometric analysis of CCR7 and CD57 by CD4+ CD45RA- T cells in a blood and a tonsil.

Fig. S2. Analysis of cytokine and proliferative responses of tonsil CD4+ T cells defined by PD-1 and CD57 expression

Representative analysis of intracellular IL-10 and IL-4 expression by tonsils sorted according to CD57 and PD-1, either immediately ex vivo, or after activation with CD2/3/28 and either IL-6 or IL-21 for two days.

Fig. S3. Induction of granzyme expression by IL-6

Purified CD57+ and CD57- subsets of tonsils all Pd-1hi were isolated by FACS sorting and stimulated with CD3/28 +/- IL-6 for 1 day then analysed for granzyme expression. Each pair of connected circles indicates the results from a single donor.

Fig. S4. Analysis of IL-21 receptor expression in CD4+ T cell subsets.

A. Representative histograms of IL-21R expression by tonsil subsets defined according to CD57+ PD-1^{hi} (orange), CD57- PD-1^{hi} (red) and CD57- PD-1^{lo} (black), and presence or absence of CXCR5 (upper and lower panels). Right panels show results from identical analysis as left panels, with the exception of omission of the IL-21R-specific antibody (fluorescence minus one, FMO). B Summary of IL-21R analysis exemplified in **A**, for CD4+ T cells from blood and tonsils, with subsets defined by CD57 and PD-1 expression.

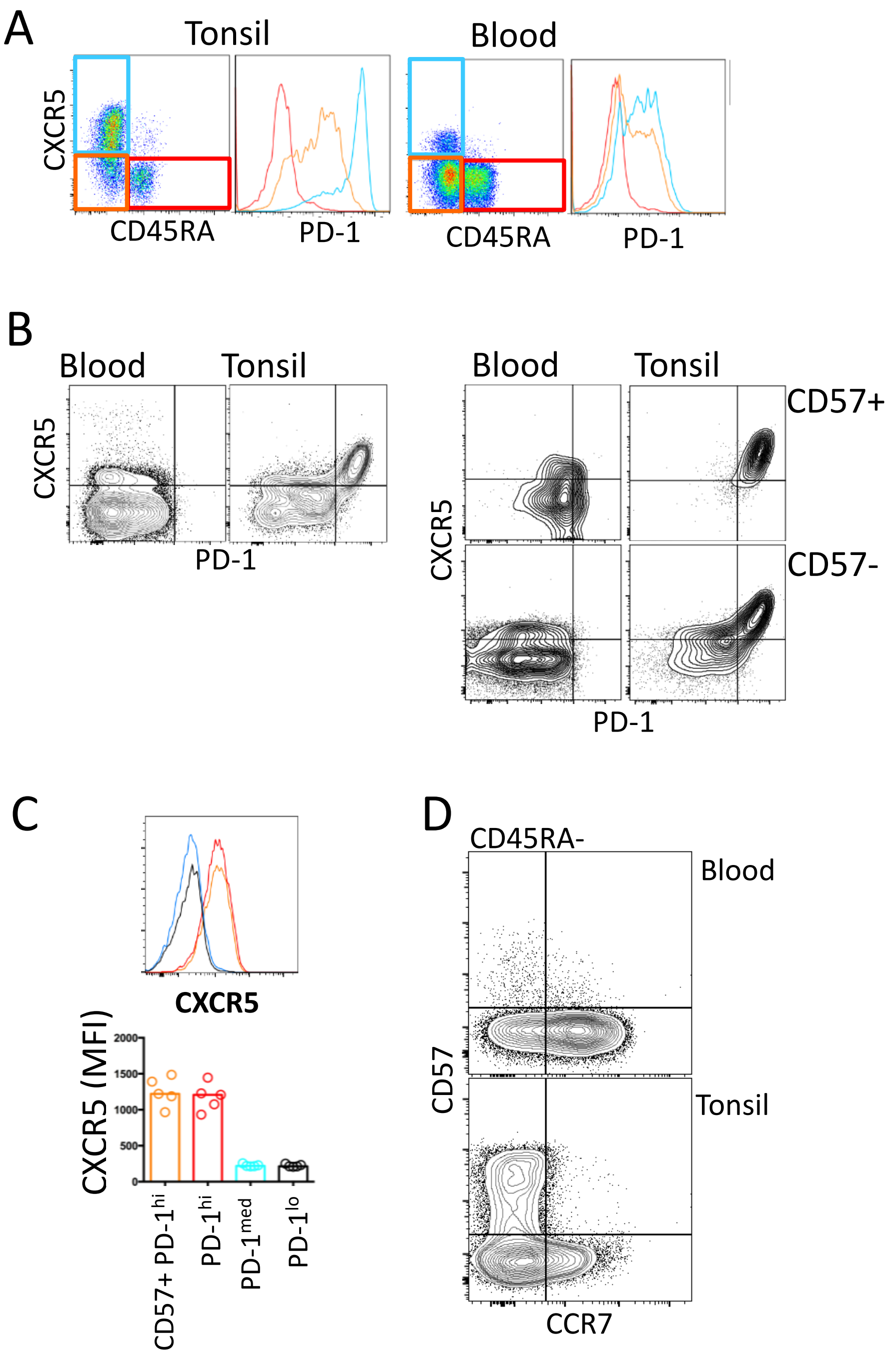

Supplementary fig. 1

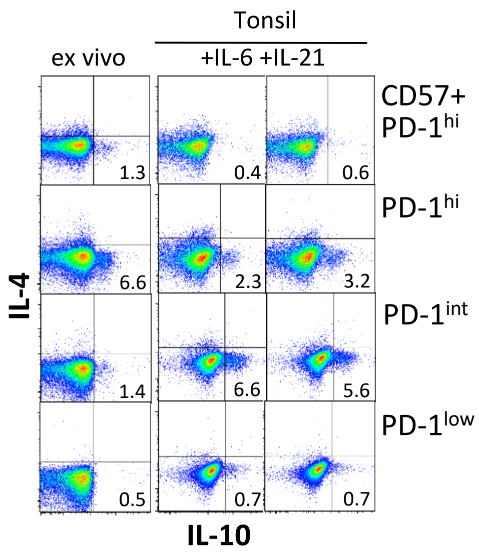

Supplementary fig. 2

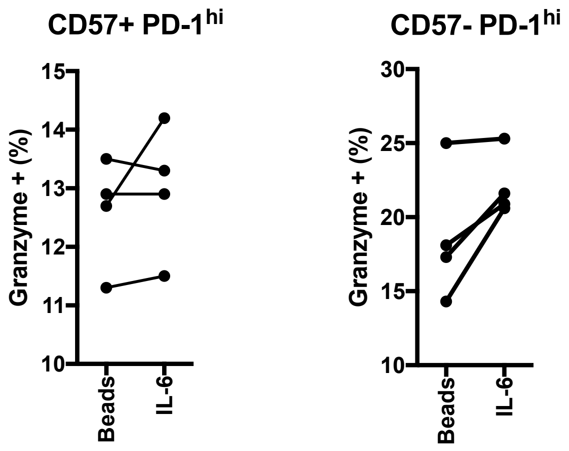

Supplementary fig. 3

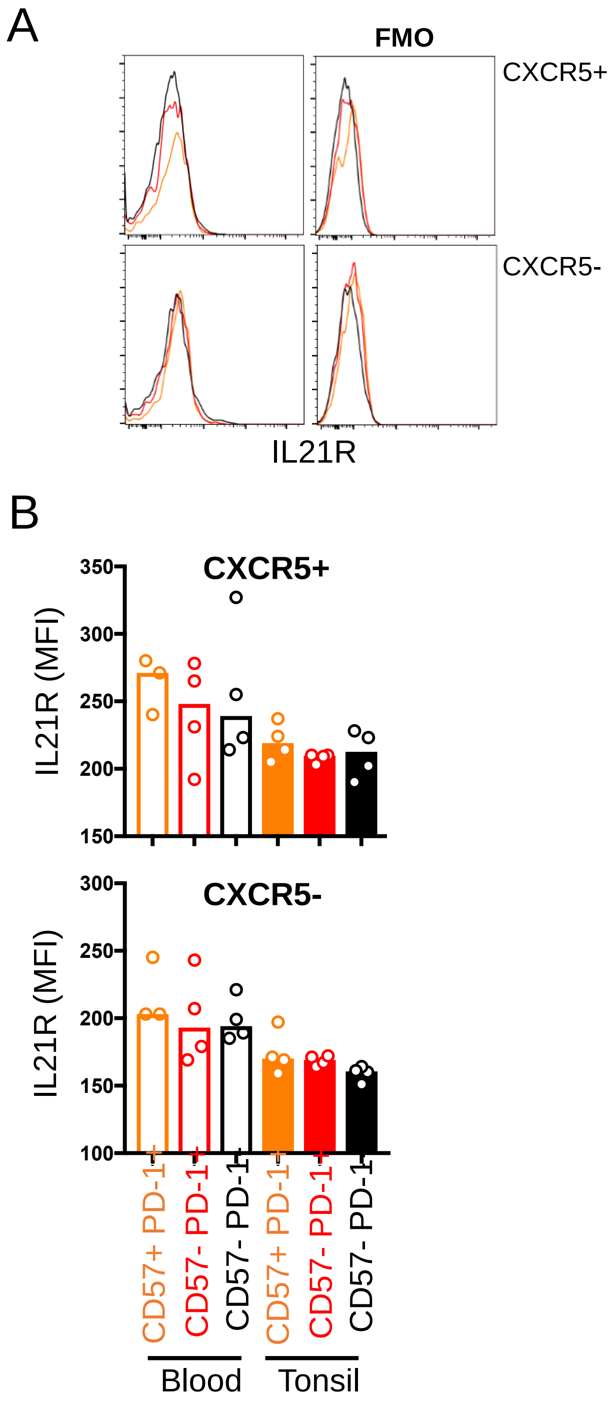
A
FMO
CXCR5+
CXCR5-
IL21R
B
CXCR5+
IL21R (MFI)
350
300
250
200
150
CXCR5-
IL21R (MFI)
300
250
200
150
100
CD57+ PD-1+
CD57- PD-1+
CD57- PD-1-
CD57+ PD-1+
CD57- PD-1+
CD57- PD-1-
Blood
Tonsil
